# Fabrication of an Anti-Reflective Microstructure on ZnS by Femtosecond Laser Bessel Beams

**DOI:** 10.3390/molecules26144278

**Published:** 2021-07-14

**Authors:** Xun Li, Ming Li, Hongjun Liu, Yan Guo

**Affiliations:** 1 State Key Laboratory of Transient Optics and Photonics, Xi’an Institute of Optics and Precision Mechanics of CAS, Xi’an 710119, China; lixun@opt.cn (X.L.); guoyan@opt.ac.cn (Y.G.); 2 University of Chinese Academy of Sciences, Beijing 100049, China; 3 Collaborative Innovation Center of Extreme Optics, Shanxi University, Taiyuan 030006, China

**Keywords:** femtosecond laser, ZnS, antireflective sub-wavelength structures (ASS), Bessel laser

## Abstract

As an important mid-infrared to far-infrared optical window, ZnS is extremely important to improve spectral transmission performance, especially in the military field. However, on account of the Fresnel reflection at the interface between the air and the high-strength substrate, surface optical loss occurs in the ZnS optical window. In this study, the concave antireflective sub-wavelength structures (ASS) on ZnS have been experimentally investigated to obtain high transmittance in the far-infrared spectral range from 6 μm to 10 μm. We proposed a simple method to fabricate microhole array ASS by femtosecond Bessel beam, which further increased the depth of the microholes and suppressed the thermal effects effectively, including the crack and recast layer of the microhole. The influence of different Gaussian and Bessel beam parameters on the microhole morphology were explored, and three ASS structures with different periods were prepared by the optimized Bessel parameters. Ultimately, the average transmittance of the sample with the ASS microhole array period of 2.6 μm increased by 4.1% in the 6 μm to 10 μm waveband, and the transmittance was increased by 5.7% at wavelength of 7.2 μm.

## 1. Introduction

In view of its excellent optical transmission in mid- and far-infrared, ZnS has critical applications in many optic components (lens, optical fibers, windows, etc.) and optoelectronic devices (solar cells, photodetectors, light-emitting diodes, etc.) [1]. However, the surface Fresnel reflection caused by refractive index mismatch at the interface between two different media, greatly impedes the transmission of light, and deteriorates the signal intensity and thermal imaging quality [2,3]. Especially for optical windows of military and aerospace applications, Fresnel reflection brings about a decrease in device detection range and strike accuracy. The traditional multi-layer thin films coated on the material exhibit good antireflective performance, but the problem of film shedding or cracking is frequently encountered owing to thermal expansion or low temperature shrinkage and mechanical grasp. More importantly, low damage threshold, narrow bandwidth, small incident angle and harsh environmental requirements are the main bottlenecks faced by coating technology [4,5,6]. Therefore, antireflective subwavelength structures (ASS) may be used instead of thin film coating to reduce Fresnel scattering and increase spectral transmittance [7,8,9], providing a continuously and linearly graded refractive index profile at the interface of substrate and air [10,11]. It has the advantages of wide bandwidth, large angular response, high mechanical strength, and surface hydrophobicity [12,13,14]. The current ASS manufacturing technologies, such as machining [15], electron beam lithography [16], lithography [17], reactive ion lithography [18], interference lithography [19] and nanoimprint lithography [7], have the shortcomings of limited materials, complex procedures, low efficiency and inability to achieve curved parts processing. Femtosecond laser is a unique technology for fabricating subwavelength structures (SWMS), on account of its simplicity, mask-less, flexible machining of space, high processing accuracy and environmental friendliness [20,21]. Unfortunately, the Gaussian beam has a concentrated energy distribution, which results in a larger microstructure roughness and significant thermal effects (recast layer, micro-cracks) on the surface of the material. Reducing the energy of the Gaussian beam is an inevitable choice to suppress the thermal effect and conform to the requirements of ASS diameter, but the depth of the ASS has to be reduced simultaneously, making the anti-reflection invalid. The researchers have used fs laser combined with other methods to fabricate ASS of transparent media. Qian-Kun Li et al. fabricated SWMS on a sapphire surface by femtosecond laser direct writing assist with wet etching, by which maximum light transmittance reached 92.5% at a wavelength of 4 μm [22]. Fan Zhang et al. proposed a three-dimensional femtosecond laser writing (3D fs-DLW) method using a three-dimensional scanning galvanometer to fabricate inverted conical and pyramidal SWMS, the maximum transmittance of which reached 85.2% at far-infrared of 9 μm [2]. Yangping Li et al. employed ps Bessel lasers to fabricate SWMS on the surface of CVD ZnS, and demonstrated that the ps Bessel lasers are more suitable than fs ones for ablating holes on ZnS [3]. Bushunov et al. adopted several methods of ASS fabrication on chalcogenide crystals, including direct single pulse ablation using 200 fs pulses, ablation with in-depth focusing, ablation in the presence of additional spherical aberration and ablation with obstruction of peripheral rays, and finally achieved 97% transmittance in the wavelength range of 2.7–8 μm [23]. However, reports of the antireflective subwavelength structures (ASS) on ZnS fabricated by fs pulsed laser are still limited. It is still a challenge to develop a simple, efficient and low thermal effect femtosecond laser direct method for preparing ASS on ZnS.

Hence, in this study, a simple method for preparing ASS on ZnS by femtosecond Bessel beams is proposed. We firstly designed the optical system of the Bessel beam, and investigated the difference of light intensity distribution between the Gaussian light and Bessel beam. Secondly, the influence of different Gaussian and Bessel beam parameters, including different pulse number and single pulse energy, on the microhole morphology were explored. In addition, the spectral transmittance and reflectance of ASS with three different periods also have been discussed in detail, and the Raman spectra of samples processed by Bessel beams were tested.

## 2. Results and Discussion

### 2.1. Optical Design and Laser Intensity Distribution of Bessel Beams

**Figure 1:**
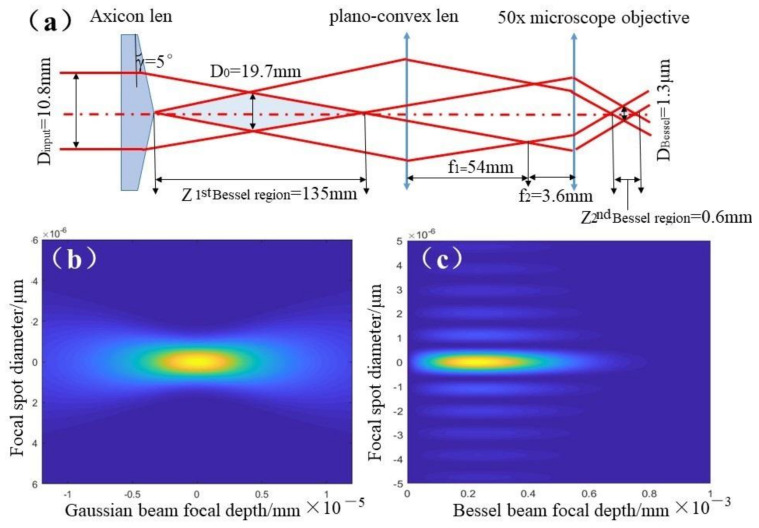
Optical system design and intensity distribution of Bessel laser beam. (**a**) The optical system design of Bessel Beam; (**b**) Laser intensity distribution of Gaussian Beam; (**c**) Laser intensity distribution of Bessel Beam.

Bessel beams can maintain their transverse shape invariant over quite long propagation distance, denoted as “diffraction-free”. Explained from the characteristics of penetration geometry, nonlinear robustness and interaction phenomenology [24,25], the zero-order Bessel beam shows its decisive advantage over the Gaussian beam in making deeper microholes in transparent materials by femtosecond laser pulses. Firstly, the Bessel beam overcomes to certain extent the strong Kerr self-focusing and plasma defocusing effects, which are conducive to extending the penetration of the laser pulse inside the bulk material. Secondly, the spot diameter of the Bessel beam was still 1.3 μm due to the microscope being 50×, but the focal depth of the Bessel beam was modulated by the 5° axicon lens and stretched to 600 μm, using a 54 mm plano-convex lens and 50× microscope objective, (Z_2nd Bessel region_), as displayed in Figure 1a. In contrast, Figure 1b depicts that the Gaussian spot diameter and focal depth of the 50× objective lens were 1.3 μm and 1.3 μm, respectively. Hence, from the perspective of geometric propagation theory, the longer focal depth of the Bessel beam is capable of processing deeper micro-holes in transparent material. In addition, the focal depth of the Gaussian point varies from 1.3 μm to 600 μm (Z_2nd Bessel region_), which reduces the laser energy density perpendicular to the optical axis by hundreds of times, as exhibited in Figure 1b,c. Making the energy density close to the damage threshold of ZnS can effectively reduce the surface roughness, on account of suppressing surface splashing and resolidification.

### 2.2. The Microhole Ablated by Gaussian Beam

**Figure 2:**
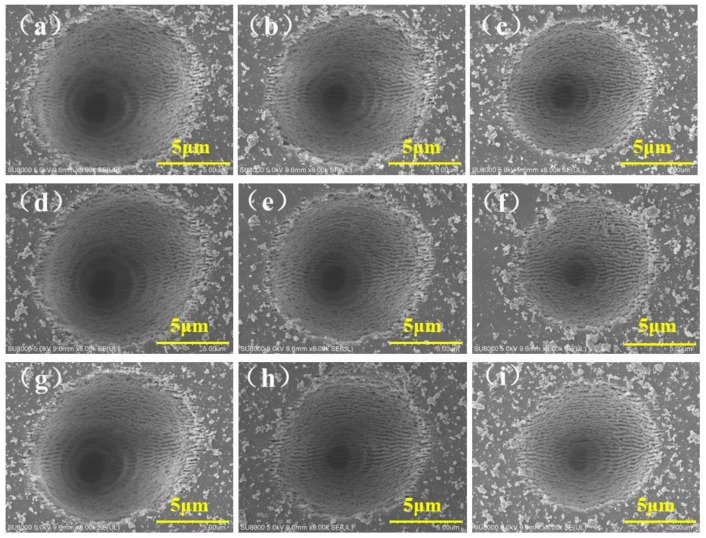
The surface morphology of microholes was ablated by Gaussian beam with different single pulse energy and pulse numbers. (**a**) The microholes were ablated with Gaussian beam at 4 μJ, and pulse number were 1 × 10^6^; (**b**) The microholes were ablated with Gaussian beam at 4 μJ, and pulse number were 1 × 10^5^; (**c**) The microholes were ablated with Gaussian beam at 4 μJ, and pulse number were 1 × 10^4^; (**d**) The microholes were ablated with Gaussian beam at 3 μJ, and pulse number were 1 × 10^6^; (**e**) The microholes were ablated with Gaussian beam at 3 μJ, and pulse number were 1 × 10^5^; (**f**) The microholes were ablated with Gaussian beam at 3 μJ, and pulse number were 1 × 10^4^; (**g**) The microholes were ablated with Gaussian beam at 2 μJ, and pulse number were 1 × 10^6^; (**h**) The microholes were ablated with Gaussian beam at 2 μJ, and pulse number were 1 × 10^5^; (**i**) The microholes were ablated with Gaussian beam at 2 μJ, and pulse number were 1 × 10^4^.

Figure 2 illustrates the microholes’ morphology of the ZnS fabricated by different Gaussian beam parameters, in which the laser single pulse energy and pulse number changed from 4 μJ to 2 μJ and 1 × 10^6^ to 1 × 10^4^, respectively. Figure 2a–i shows that with the decrease of laser single pulse energy and repetition frequency, the diameter of the microhole became smaller, which was caused by the reduction of the laser incident fluence accumulated on the surface of the material. In addition, it reveals that the diameter of the microhole processed by the Gaussian beam was much larger than that of the Bessel beam under the same laser parameters. The diameters of the microholes were all more than ten microns since the Gaussian beam has much higher energy density than the Bessel beam. When the diameter of microhole was reduced to 1–2 μm, it severely limited the accessible depth [26], which makes the surface equivalent refractive index change little with the depth, causing the unsatisfactory anti-reflection effect of the subwavelength microstructure. In addition, after being cleaned by hydrofluoric acid corrosion, there were still recast layers and splashes on the surface of the sample. These kinds of recast layers and splashes accumulated more seriously when preparing microhole arrays of ASS. This was because the Gaussian spot had a very high laser energy density, much higher than the damage threshold of the ZnS material, resulting in violent ablation of the sample. The molten material in the hole was ejected, adhered, and deposited on the surface of the sample. In particular, there were periodic fringes on the bottom and side walls of the hole, just as for the microhole fabricated by a Bessel beam. This phenomenon will be discussed in detail in Section 2.3. The above-mentioned significant thermal effect will increase the roughness of the microstructure, which is not conducive to the increase of the transmittance of ASS.

### 2.3. The Microhole Ablated by Fs Bessel Beam

**Figure 3:**
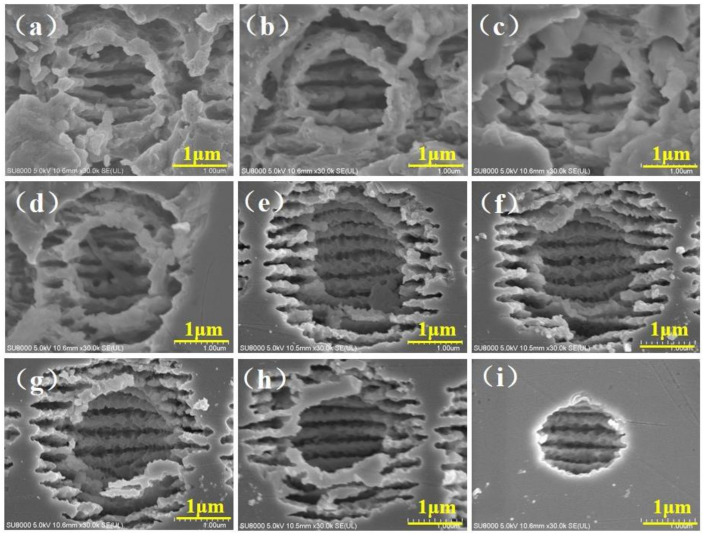
The surface morphology of microholes was ablated by Bessel beam with different single pulse energy and pulse numbers. (**a**) The microholes were ablated with Bessel Beam at 4 μJ, and pulse number were 1 × 10^6^; (**b**) The microholes were ablated with Bessel Beam at 4 μJ, and pulse number were 1 × 10^5^; (**c**) The microholes were ablated with Bessel Beam at 4 μJ, and pulse number were 1 × 10^4^; (**d**) The microholes were ablated with Bessel Beam at 3 μJ, and pulse number were 1 × 10^6^; (**e**) The microholes were ablated with Bessel Beam at 3 μJ, and pulse number were 1 × 10^5^; (**f**) The microholes were ablated with Bessel Beam at 3 μJ, and pulse number were 1 × 10^4^; (**g**) The microholes were ablated with Bessel Beam at 2 μJ, and pulse number were 1 × 10^6^; (**h**) The microholes were ablated with Bessel Beam at 2 μJ, and pulse number were 1 × 10^5^; (**i**) The microholes were ablated with Bessel Beam at 2 μJ, and pulse number were 1 × 10^4^.

Figure 3 illustrates the microholes’ morphology of ZnS fabricated by different Bessel beam parameters, in which the laser single pulse energy and pulse number altered from 4 μJ to 2 μJ and 1 × 10^6^ to 1 × 10^4^, respectively. When the pulse energy was 4 μJ, the energy density of the zero-order and diffraction rings were much higher than the material damage threshold, resulting in explosions and violent shock waves in and around the microholes. The severe recast at the edge and the annular area of the hole, which was formed by spraying, adhering, depositing, and cooling the molten material in the cavity to the edge of the hole during the ablation process, are shown in Figure 3a. As the number of pulses were reduced from 1 × 10^5^ to 1 × 10^4^, even though the incident accumulated fluence of the material was reduced, the effects were still higher than the material damage threshold. The melting and recasting phenomenon was not well controlled, as displayed in Figure 3b,c. Figure 2f and Figure 3d show the microholes’ morphology with the single pulse energy 3 μJ; as the number of pulses decreased from 1 × 10^6^ to 1 × 10^4^, the microhole evolved to a clear morphology in virtue of the suppression of thermal effects, yet processing areas of the first-order Bessel spot and the first-order diffraction ring still overlapped. As single pulse energy was decreased to 2 μJ, the area of the zero-order diffraction microhole and the first-order diffraction ring could be distinguished, the surrounding ring still existed, as presented in Figure 2h and Figure 3g. When the pulse number dropped to 10,000, a single hole without surrounding rings and with a clean morphology was obtained as shown in Figure 3i, due to the fact that the intensity of the diffraction ring of the Bessel laser was not strong enough to ablate the material.

It is worth noting that there were periodic fringes on the bottom and side walls of the hole, on account of the interference effect between the incident laser and excited surface wave during femtosecond laser-material interactions [27,28]. Wavefront interference fringes can be generated where the surface wave captures electrons more strongly. The nanostructures’ morphology of the nanoripples evolves with the repetition rate, beam polarization, and laser power [1]. In particular, only a large number of pulses can maintain a plasma standing wave, which can modulate the electromagnetic field on the surface of the sample to produce nanostructures, and for pulses with lower repetition rates, such nanostructures may not emerge [29,30]. Furthermore, the orientation of the nano-ripples is always perpendicular to laser polarization, which originates from the multi-beam laser interference effect and laser local field enhancement [1,27]. Consequently, the 10,000 laser pulses used in this experiment can easily form periodic nanostructures at the bottom of the micropores. In addition, the directions of the nanoripples induced by different repetition frequencies and pulse energies were the same since the laser linear polarization state had not changed.

### 2.4. The ASS Microhole Array Ablated by Bessel Beam

**Figure 4:**
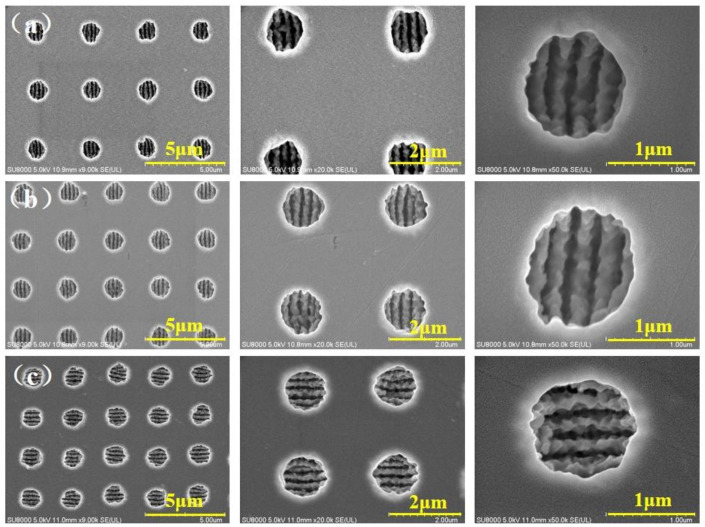
SEM images of microhole arrays with different periods. (**a**) 3.6 μm period; (**b**) 3.0 μm period; (**c**) 2.6 μm period.

**Figure 5:**
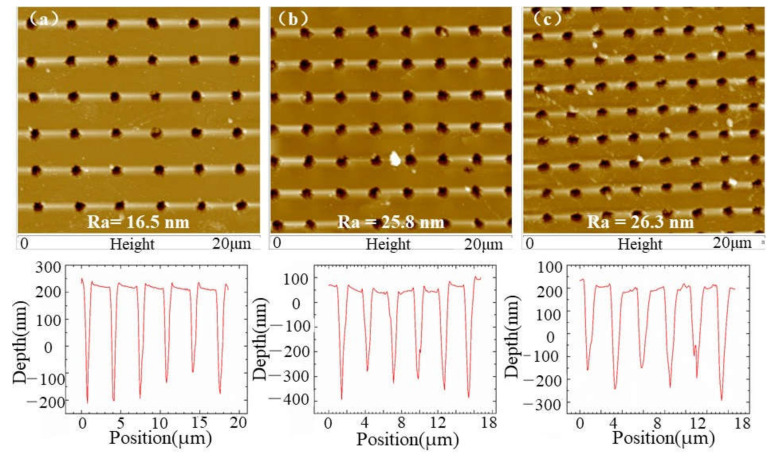
AFM images of microhole arrays with different periods. (**a**) 3.6 μm period; (**b**) 3.0 μm period; (**c**) 2.6 μm period.

By studying the effect of ASS period on the antireflection effect intuitively and intensively, three kinds of ASS with different periods changing from 3.6 μm to 2.6 μm were fabricated by Bessel beam, as displayed in Figure 4a–c. The recast layer and cracks on the bottom and side wall of microholes were apparently suppressed, and the areas between the microholes were still smooth without splashes or cracks. Figure 5a–c lists AFM images of the ASS on ZnS with average depth of 0.5 μm. It demonstrates that the Bessel beam has experienced undesired nonlinear effects before it converges on the axis, including Kerr self-focusing, multiphoton ionization, avalanche ionization, plasma volume shielding, defocusing, etc., which affect the normal transmission of the beam [31]. In addition, the surface of ASS measured by AFM is relatively smooth with surface roughness of 16.5 nm and 26.3 nm in a square region of 20 × 20 μm. The smooth surface primarily benefits by Bessel laser with a small heat-affected zone [14], which can reduce light scattering to enhance the transmittance.

**Figure 6:**
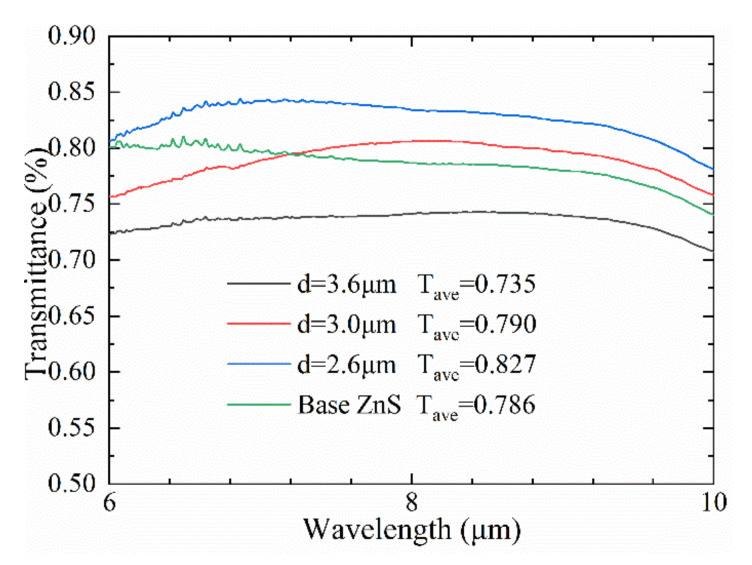
IR transmittance of the ZnS before and after ablating microhole array.

Figure 6 demonstrates the IR transmittance of the ZnS before and after ablating the microhole array. It verifies that the average transmittance of ASS microhole arrays with different periods was completely different. Firstly, compared to the value of 78.6% in base ZnS, the average transmittance of the ASS microhole array with period of 2.6 μm increased by 4.1% over the 6–10 μm wavelength range, and the highest light transmittance value of 84.28% reached at wavelength of 7.2 μm. The increase of transmittance was basically attributed to the ASS on the surface having enough depth to introduce a graded refractive index between *n* = 1 (air) and *n* = 2.2 (ZnS), which improved Fresnel scattering at the interface between material and air. More importantly, the Bessel beam reduced the energy density of the Gaussian beam along the optical axis by almost five hundred times to suppress surface splashes and resolidification, which can effectively reduce surface roughness. Low roughness can inhibit the scattering of infrared light on the inner wall of the microhole, which is beneficial to raise the infrared transmission light intensity of the microstructure. Moreover, the average spectral transmittance of ASS decreased by 5.1% with a period of 3.6 μm in a broadband from 6 μm to 10 μm. The subwavelength period restrictive condition for perfect antireflection can be deduced from a grating diffraction equation; nonzero diffraction orders disappear in SWMS, and the constrained equation of period with normal incidence light is derived from [1]:

(1) $$\frac{d}{\lambda }<\frac{1}{n_{2}+n_{0}}$$

where *d*, $n_{2}$, and $n_{0}$ are period, refractive index of base material, and refractive index of air separately. According to the formula calculation, the period of the ASS must be less than 3.1 μm, otherwise most of the incident infrared light will be scattered by the microholes array, attributed to the fact that the high-order scattered light becomes stronger, and the zero-order diffracted transmission light gets weaker.

The transmittance of the microhole array with a period of 3 μm showed more interesting phenomena. The spectral transmittance of ASS was lower than that of base ZnS substrate in a broadband from 6 μm to 7.2 μm, but rose by 1.6% in the wavelength of 7.2 μm to 10 μm effectively, and in particular obtained a maximum transmittance of 80.24% at a wavelength of 8.2 μm. As demonstrated in Equation (1), the center wavelength of ASS λ is 9.6 μm; the far-infrared transmittance of the ASS was therefore improved compared to that of the ZnS substrate. The transmittance decreased with a wavelength of less than 7 μm, on account of the fact that the period of 3.0 μm was too close to the wavelength of the incident light, which does not meet the conditions of subwavelength antireflection characteristics. Finally, in the 7.2 μm to 10 μm wavelength range, the 3 μm period ASS has a relatively large refractive index step due to the larger fill factor [2], and thus its transmission was slightly worse than the ASS with a period of 2.6 μm. Furthermore, the uneven nanostructure created on the bottom and side wall of microholes increased the surface roughness, enhancing the Fresnel scattering of the light beam inside the micropore, which reduced the transmission of the infrared incident light.

**Figure 7:**
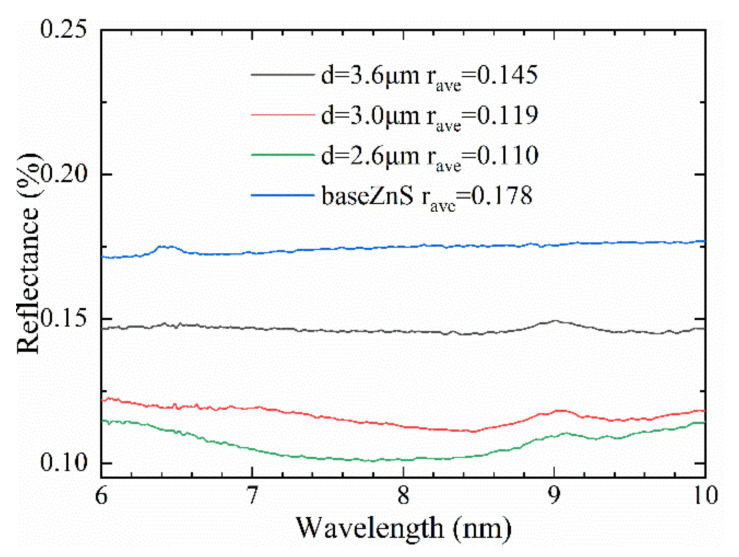
IR reflectance of the ZnS before and after ablating microhole array.

Figure 7 demonstrates the reflectivity of the ASS with period of 3 μm and 2.6 μm reduced by 5.9 % and 6.8%, respectively. The surface microstructure depressed the infrared reflectance of the ZnS surface visibly; some energy was converted into the intensity of infrared transmitted light, the other part was absorbed, which included the resonance absorption by the nanoripple, the multiple scattering and absorption by nanoparticles [31]. The result shows that laser-induced periodic nanostructures can effectively improve the absorption of light and reduce the reflectivity of the material. Firstly, the surface nanostructure layer forms a gradient refractive index between the air and the substrate, which improves the light trapping effect and helps to capture more light into the micropores. Furthermore, the nanostructure of the microhole increases the surface roughness and enhances the Fresnel scattering effect of the entering light. In addition, the laser-induced periodic distribution of nanoripple is beneficial for broadening surface plasmon resonances, resulting in more efficient light absorption [32].

**Figure 8:**
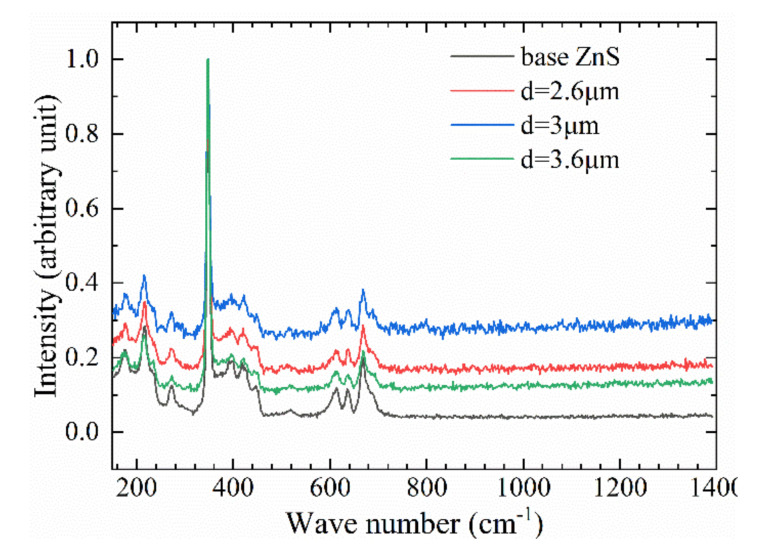
Raman Spectroscopy of ZnS Surface.

The Raman characteristic peaks displacement of ZnS before and after femtosecond laser processing was basically the same; 350 cm^−1^ [33] corresponds to the longitudinal optical vibration mode (LO) of ZnS as displayed in Figure 8. The difference is that the surface microstructure weakens the Raman scattering signal, causing the entire Raman curve to move downward. Meanwhile, the Raman characteristic peak intensity of ZnS treated with Bessel beam had changed, and its structure was relatively stable without forming a new crystal structure, such as ZnO, and no photooxidation reaction occurred. It also showed that the infrared transmission spectrum change of the ZnS surface was completely determined by the size of the microstructure, rather than the effect of the surface oxide.

## 3. Materials and Methods

**Figure 9:**
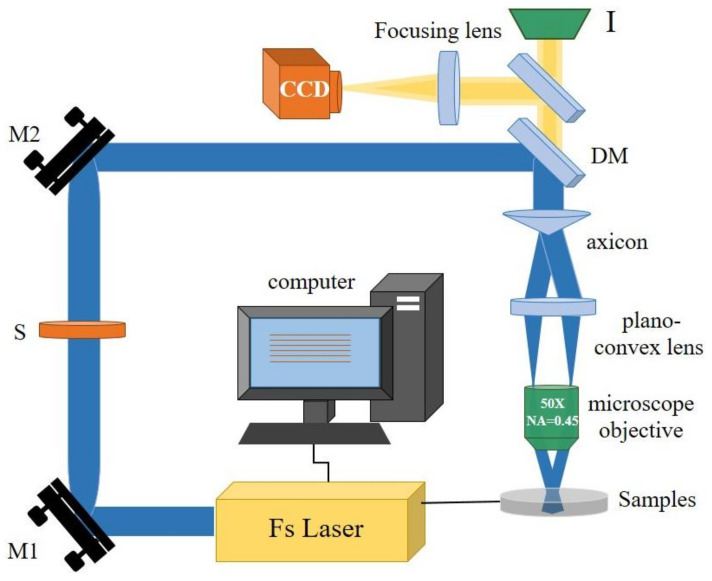
Experimental set-up of femtosecond laser Bessel beam processing system.

ZnS samples of φ25.4 × 2 mm were polished to an optical grade without coating. Figure 9 shows the schematic of the fs laser Bessel beams processing system. In the experiment, an industrial high repetition frequency femtosecond laser (Pharos, lightconversion, Vilnius, Lithuania) with linearly polarized pulse light was used. Its wavelength, pulse width, and repetition rate are 1030 nm, 290 fs, and 1–1 MHZ adjustable, respectively. The original output beam of the laser source was a Gaussian beam and could be shaped into a Bessel beam through an axicon. The sample was installed on a 3D high-precision adjustment frame (TSD-801CDM, Sigma, Kanagawa, Japan) to level it accurately, and then the 3D high-precision adjustment frame was mounted on a three-axis motion platform (P-622.2CD, PI) with a movement repeatability of 2 nm. The sample was moved along the X/Y axis by a distance of L that is the period of the array. The Bessel beam was formed by 5° axicon (AX255-B, Thorlabs, Newton, NJ, USA), and squeezed to a focal depth of 600 μm (focus spot of 1.3 μm) by a beam compressor, which consists of 54 mm plano-convex lens and 50× microscope objective (NIR-50-45-P, Tokyo, Japan). The surface morphology of the ASS was characterized by a scanning electron microscope (SEM, SU8010, Hitachi, Tokyo, Japan). The profiles of the structures were observed by an atomic force microscopy (AFM, Innova, Bruker, Leipzig, Germany) in tapping mode. Then, the infrared transmittance of the samples was measured by an infrared microscope (FTIR, Bruker, vertex70+Hyperion1000, Leipzig, Germany). The evolution of the crystal structure of the sample material was tested by a Raman spectrometer (confocal Raman microscope, Via Qontor, Renishaw, London, UK).

## 4. Conclusions

In summary, a simple method of micro-hole array ASS fabricated by Bessel beams was proposed. In order to overcome the shortcomings of traditional Gaussian beam manufacturing SWMS, we firstly designed the optical system of the Bessel beam, and explored the difference of light intensity distribution between the Gaussian light and Bessel beam. This indicated that the Bessel beam further increased the depth of the micro-holes slightly and suppressed the thermal effects effectively including cracks and recast layer on the edges of the microhole. In addition, the influence of different pulse numbers and single pulse energy of Bessel beams and Gaussian beams on the microhole morphology was studied separately, and finally a clean morphology hole without surrounding first order diffraction rings was obtained under 2 μJ single pulse energy and 10,000 pulses by Bessel beam. Furthermore, three ASS structures with different periods were fabricated using the optimized Bessel parameters, and the average transmittance of the ASS microhole array with a period of 2.6 μm increased by 4.1% in the far-infrared range of 6 μm to 10 μm, and increased by 5.7% at the wavelength of 7.2 μm. Finally, the Raman test of the micro-hole array after Bessel beam treatment showed that the increase in spectral transmittance was only related to the microstructure characteristics, and had nothing to do with the oxidation change of the sample.

## Data Availability

Not applicable.

## Sample Availability

Samples of the compounds ZnS are available from the authors.
